# Influence of Personal Traits, Social Relationships, and External Resources on the Development of Emotional Resilience in Children From East London: Protocol for an Observational Accelerated Longitudinal Cohort Study

**DOI:** 10.2196/70797

**Published:** 2025-07-08

**Authors:** Francois van Loggerenberg, Milena Nikolajeva, Daniele Porricelli, Imogen Hensler, Aisling Murray, Eleanor Keiller, Julia Michalek, Dennis Ougrin, Jennifer Y F Lau

**Affiliations:** 1 Youth Resilience Unit Wolfson Institute of Population Health Queen Mary University of London London United Kingdom; 2 Parc Sanitari Sant Joan de Déu Docència i Innovació Unitat de Recerca Barcelona Spain

**Keywords:** mental health, school, primary school, scholar, student, feasibility, child, mental well-being, youth

## Abstract

**Background:**

Emotional resilience is a dynamic process by which individuals may prevent, overcome, and thrive following challenging events. Emotional resilience can be defined as absence of negative outcomes (ie, symptoms of psychopathology) and/or the presence of adaptive outcomes (ie, well-being). Despite the wealth of research tracking the nature of and contributions to emotional resilience in adolescence and adulthood, there is a dearth of evidence on the nature of resilience and its development during preadolescent childhood despite this being an important preventative period for later mental health difficulties and a period when emotional experiences change.

**Objective:**

Our primary study objectives are 3-fold: to explore how preadolescent children growing up in deprived areas of London may operationalize resilience, evaluate whether there are differences in the development or trajectories of resilience among our target population, and understand what contributes to resilience pathways over time. Additionally, our research aims to better understand the psychometric properties of resilience measures used in a preadolescent sample and assess the feasibility of developing a longitudinal cohort study of preadolescent children in East London.

**Methods:**

We will conduct an accelerated longitudinal cohort study in primary schools across the broad geographical area of East London. The multimethod approach will span across 3 data collection arms: (1) child cognitive tasks and psychometric questionnaires in classroom settings, (2) teacher ratings, including teacher assessments of school mental health provisions, and (3) parent questionnaires. We aim to recruit approximately 1200 children aged 7-11 years at baseline across UK school years 3, 4, and 5. Our measures will span themes of resilience and mental health, as well as personal, social, and community resources available to the children. We will collect quantitative data via questionnaires from children, their parents, and school staff. We will collect qualitative data from the children through paper-based tasks.

**Results:**

Study recruitment commenced in October 2022 and continued till December 2023. Baseline testing commenced in October 2022 and continued till December 2023; 873 students were enrolled at baseline. Follow-up is anticipated to continue at least annually until June 2027.

**Conclusions:**

This study will assess the feasibility of conducting a longitudinal cohort study on preadolescent children in East London. Alongside evaluating the psychometric properties of resilience measures used in this age group, this study will explore how resilience develops in children across time and relate this to other outcome measures. By identifying how personal, social, and community resources may affect resilience in preadolescent children, we will enhance the understanding of how emotional resilience develops in preadolescent children, and future studies will be able to develop interventions to boost resilience by targeting young and diverse populations.

**Trial Registration:**

ISRCTN ISRCTN12430839; https://www.isrctn.com/ISRCTN12430839

**International Registered Report Identifier (IRRID):**

DERR1-10.2196/70797

## Introduction

### Background

Resilience is a dynamic process that refers to the capacity of individuals to prevent, overcome, and even thrive following the experience of challenging events [1]. Resilience outcomes can be captured through functioning in different domains, but here, we focus on emotional resilience, which can reflect the presence of an adaptive outcome (well-being, life satisfaction) or the absence of a negative outcome (mental illness) [2] following the experience of stress (internal or external), which can range from adverse childhood events to ongoing chronic stressors. Resilience can be conceptualized as an individual characteristic (eg, grit, perseverance, determination), but it also includes opportunities to harness and navigate access to culturally relevant external resources (eg, contact seeking, participation in community groups) [3]. Although there is a growing body of literature measuring and tracking emotional resilience (good mental well-being and absence of psychopathology following stress) and the factors and resources that contribute toward it in adolescence [4,5] and young adulthood [6,7], there is notably less research on the nature of resilience and how and when it develops at even earlier stages of development such as preadolescent childhood. This phase of development is important because efforts to enhance resilience here have preventative potential; children’s emotional experiences are also changing, which may make them more receptive and amenable to acquiring, learning, and consolidating lifelong adaptive strategies for managing emotions.

A study of emotional resilience as it emerges across middle and late childhood into early adolescence and the factors that predict early resilience trajectories would be an invaluable addition to the field for several reasons. First, early signs of reduced emotional resilience could predict reduced well-being and increased mental health problems and their impact in youth, enabling us to identify those who are less well-equipped to manage the challenges and opportunities of adolescence—and in turn, across the lifespan. Second, understanding the factors contributing toward the development of emotional resilience could help with the development of preventative programs and the planning of resources to reduce negative outcomes across the lifespan. Finally, as resilience reflects “the capacity of a dynamic system to adapt successfully to disturbances that threaten system function, viability, or development,” it is likely that its nature and core components vary in childhood compared to adolescence and adulthood. More specifically, preadolescent childhood is a period when emotional experiences are becoming more complex, varied, and at times, more intense [8]. These age-based differences may mean that the factors contributing to resilience change between childhood and adolescence, as they begin to stabilize and consolidate. Yet, these more enriched experiences may also allow a more plastic period for children to learn and practice adaptive strategies for managing stronger, more intense emotional experiences.

A better understanding of emotional resilience in mid-to-late childhood would therefore allow us to propose developmentally sensitive theories of resilience. The Youth Resilience Unit is based at Queen Mary University of London—in the diverse and relatively economically deprived area of East London. The university has a commitment to its location and surrounding community, and it is an area that is underresearched; therefore, it has a focus in terms of understanding the development of resilience in this often challenging sociocultural environment. In the 2021 census, East London was found to be much more culturally diverse in relation to the whole of England and Wales, with a much higher proportion of Asian (32.6% vs 9.3%), Black (14.6% vs 4%), mixed (5.6% vs 2.9%), and other (5.1% vs 2.1%) ethnicities and significantly lower in those who identified as White (42.1% vs 81.7%) [9]. In terms of economic activity, East London also has higher levels of self-employment or no employment (10.1% vs 7.9%), students with no employment (9% vs 6.3%), unemployed (4.7% vs 2.8%), and with much lower proportion of retirees (8.6% vs 21.6%). Having a young and the most diverse population in the United Kingdom while also being historically underserved in research makes East London the perfect location for the timely addition of a new cohort study.

The Development of Emotional Resilience (DEER) study is a new (accelerated) longitudinal cohort of preadolescent children (aged 7-11 years at recruitment) residing in East London to address questions relating to (1) the early development of resilience, (2) differences across individuals in resilience trajectories, and (3) factors/resources that contribute toward resilience trajectories.

### Objectives

We have 3 primary objectives in the DEER study. These are to explore how preadolescent children growing up in deprived areas of London may operationalize resilience, evaluate whether there are differences in the development or trajectories of resilience among our target population, and understand what contributes to resilience pathways over time. Additionally, our research aims to better understand the psychometric properties of resilience measures used in a preadolescent sample and assess the feasibility of developing a longitudinal cohort study of preadolescent children in East London.

## Methods

### Recruitment and Study Design

#### Study Design

The DEER study is an observational cohort study that uses an accelerated longitudinal design. An accelerated longitudinal design recruits participants from different aged cohorts and follows them up [10], allowing both a within-time assessment of cross-age differences (cross-sectional age effect) and a cross-time assessment of within-person differences (longitudinal time effect). We will aim to enroll approximately 1200 children across UK school years 3, 4, and 5 (aged 7-11 years at recruitment) attending primary schools in East London. Children will be assessed at least once a year until they reach the end of their first term of secondary school. Parents/carers and teachers will be asked to complete questionnaires to complement children’s self-reported measures annually.

#### Settings and Participants

Schools were contacted from a database containing all schools located in the broad geographical area of East London. Of the schools approached, a total of 10 primary schools in East London across 5 boroughs were recruited. These boroughs (Waltham Forest, Havering, Redbridge, Newham, and Tower Hamlets) include among the most densely populated, socioeconomically, and ethnically diverse areas in London [11].

#### Consenting Process

Prior to enrolment, research assistants conducted an in-school talk aimed at children from the targeted age group about the DEER study. After the talk, they distributed information sheets to children and their parents/carers. Initially the ethics committee recommended that the consenting process be opt in, that is, in addition to assent collected from children, parents or guardians needed to provide written consent in order for a child to take part. When it became apparent that enrolment was very low (by November 2022, only 6.9% (131/1898) of the consent forms were returned signed) and potentially biased in favor of parents and families who were better resourced, the ethics committee allowed for an opt out process. This means that for the 10 recruited schools, 6 are opt-in only, 1 began with opt-in processes but then agreed to participate using opt-out procedures, and the remaining 3 are opt-out only schools. For opt-out schools, parents were asked to return a form or contact the school or research team to indicate they did not want their child to take part, and then no data were collected from the opted-out children. The team liaised with schools to ensure that only students who met our inclusion criteria were enrolled in the study. Because most of the data are self-reported, some of our measures are not suitable for students who have been diagnosed with an intellectual disability disorder.

#### Participant Inclusion Criteria

In order to be enrolled in the study, children must be (1) attending a primary school in East London, (2) in school years 3 to 5 (at recruitment), (3) able to understand written and spoken English, (4) willing and able to provide assent, and (5) able to obtain parental/carer consent for opt-in schools.

#### Participant Exclusion Criteria

To participate in this study, children must not be diagnosed with an intellectual disability disorder.

#### Sample Size

Based on the planned analyses, the target recruitment was 1200 children in school years 3-5 (age 7-11 years). This sample size provides (1) 94% power to detect correlations as small as 0.10 (2-tailed), *P*<.05 between resilience measures and correlates and (2) the opportunity to compare groups high and low on a psychological or social factors (eg, top and bottom 20% of the sample on attention control) or on a resilience measure and to detect even small effects (eg, Cohen *d*=0.30) at 90% power, 2-tailed, *P*<.05.

Importantly, this sample size will enable us to use novel data-driven analytic approaches to identify subpopulations with different resilience trajectories across time and to assess the predictive value of different psychosocial variables (main effects, interactions, mediation effects) on resilient trajectories by using multinomial logistic regression. This will also allow us to use other advanced quantitative analyses, including structural equation modelling (to explore mediation and moderation effects), a combination of exploratory and confirmatory factor analysis (to verify the factor structure of selected questionnaires), and network analysis to explore associations between individual items of questionnaires.

### Ethical Considerations

This study has been reviewed and approved by the Queen Mary University of London research ethics committee (QMERC22.251) on June 27, 2022. The study was prospectively registered in the International Standard Randomized Controlled Trial Number registry (ISRCTN14396374). Written assent from the children was obtained prior to any study procedures being conducted, and written consent was obtained from parents/carers if children were recruited via opt-in procedures. All participating schools gave written consent for participation via the school principals. To ensure anonymity, study data are stored in a deidentified format. Names and other identifying information (eg, addresses, contact details) are replaced by a unique participant ID number. To reduce the risk of disclosure, participant identifying information is stored separately from the research data, which will be saved on a secure server at the Youth Resilience Unit, hosted by Queen Mary University of London. To say thank you to the child’s class for taking part, we offer participation in an interactive science show organized by the Centre of the Cell, Queen Mary University of London. The show is aimed at children in primary schools and is educational and fun. Whether the show is offered at the school or the school attends the Centre of the Cell is decided by the school. We compensate parents who complete annual questionnaires £10 (US $13.74) each time.

### Procedures

Schools provided the team with students who met the inclusion and exclusion criteria and the students who the teachers felt would struggle to complete measures in a group setting. During data collection visits, researchers provide further information about the DEER study to pupils, and pupils provide written assent before completing a battery of validated questionnaires. All assessments intended for the child to complete are done in the classroom, mainly on an electronic tablet or pen and paper depending on the task, in small groups but with privacy. As the baseline assessment took up to 4.5 hours to complete, site visits were often split up across multiple sessions. Detailed information was collected about the demographics as well as the social, personal, and community factors affecting resilience in children. Trained researchers were present throughout all sessions to explain procedures and answer questions. All baseline questionnaires were administered between October 2022 and December 2023. Follow-up will be conducted at least annually until the first term of the secondary school (where possible). Parents or carers complete a set of web-based questionnaires for each year their child is enrolled in the study, for which they received financial reimbursement in the form of e-vouchers worth £10 (US $13.74). Teachers are also asked to complete paper questionnaires for each pupil enrolled in the study and to describe the provision of mental health services at the school.

### Measures

#### Design of Measures

Most outcome measures are completed via tablet computers by using data management applications (eg, Qualtrics, Research Electronic Data Capture [REDCap]). Qualitative measures are completed on paper. A paper-based case report form is used to keep track of the completion of measures for each participant. This study takes place in schools with children, and so the assessment schedule may need to be adapted to fit the practical demands of the environment. As this is a longitudinal cohort, some measures may be dropped, and new measures may be introduced in response to new knowledge, new substudies, or changes to the factors of interest. This may include changing measures that children report to be too time-consuming or onerous to complete within the allocated time. All changes to the measures will be reviewed and approved by the ethics committee prior to implementation.

#### Measures About Resilience and Mental Health

Table 1 [12-24] shows the measures we used to analyze resilience and the mental health of the children.

**Table 1:** Measures about resilience and mental health.

Name of the scale	Brief description	Style of administration
Demographics	We will be asking children to report their gender, age, and height and weight will be measured	Tablet
The Stirling Children’s Well-Being Scale [12]	Self-report questionnaire used to assess children’s emotional and psychological well-being	Tablet
The University of California Los Angeles 3-Item Loneliness Scale adapted to 10-15-year old children and Office for National Statistics, United Kingdom, 1-item Direct Measure of Loneliness [13,14]	Self-report questionnaire used to measure feelings of loneliness and social isolation	Tablet
The Revised Child Anxiety and Depression Scale [15]	Self-report questionnaire providing an overall internalizing score and subscores assessing anxiety (social phobia, panic disorder, separation anxiety, generalized anxiety, obsessive-compulsive symptoms) and symptoms of depression	Tablet
The Children’s Revised Impact of Event Scale [16,17]	A self-report questionnaire designed to screen for posttraumatic stress symptoms in children	Tablet
Body Mapping protocol [18]	Body mapping, an arts-based research method, adapted for use to understand how children make sense of their own stress and resilience	Pen and paper
Connor-Davidson Resilience Scale [19]	Self-report questionnaire assessing resilience	Tablet
The Revised Child and Youth Resilience Measure [20]	Self-report questionnaire assessing resilience	Tablet
The Student Life Satisfaction Scale [21,22]	Self-report questionnaire used to gauge an overall level of life satisfaction among children	Tablet
The Somatic Complaints List [23]	Self-report psychosomatic questionnaire used to identify how often children experience and feel pain	Tablet
The Self-Perception Profile for children [24]	Self-report questionnaire used to measure a school-age child’s sense of general self-worth and self-competence in the domain of academic skills (scholastic competence, social competence, athletic competence, physical appearance, behavioral conduct, and global self-worth)	Tablet

#### Measures About Resources, Psychological, and Social Factors

Table 2 [25-42] shows all the measures used for measuring the psychological factors, social factors, and the resources available to children.

**Table 2:** List of measures used for measuring the psychological factors, social factors, and resources available to children.

Name of the scale	Brief description	Style of administration
Short version of The Cognitive Emotion Regulation Questionnaire [25,26]	Self-report questionnaire assessing emotional regulation and cognitive copying strategies (self-blame, other blame, acceptance, planning, positive refocusing, rumination or focus on thought, positive reappraisal, putting into perspective, and catastrophizing).	Tablet
Social connectedness questionnaire [27]	A questionnaire exploring children’s global social connectedness, within the domains of family, school, peer, and community connectedness.	Tablet
The Advanced Theory of Mind battery [28]	Selected tasks from a task battery used to measure children’s ability on social reasoning and recognizing transgression.	Tablet
The Children’s Leisure Activities Study Survey [29]	Self-report questionnaire used to gain an understanding about participants’ engagement with leisurely activities, hobbies, and exercise.	Tablet
The Youth Spirituality Scale [30]	Self-report questionnaire assessing children’s connections to God or higher power as well as their relationship with religious practices. For this purpose, we will look at the Relationship with God and Religious Practices subscales only.	Tablet
The Ambiguous Story Paradigm [31]	A task used to assess children’s tendencies to select negative over benign interpretations of ambiguous situations.	Tablet
The Adapted Prospective Imagery Task [32]	A task used to assess children’s capacity to imagine past and future positive and negative events in detail.	Tablet
Self-Esteem Matrix Task [33,34]	The task is used to measure how quickly participants can find an accepting face (smiling face) in a matrix of 15 rejecting faces. Slower reaction times on this task may suggest higher sensitivity to social threats.	Tablet
The Five Field Map [35]	Self-report questionnaire used to gauge the size of the children’s social network as well as to uncover more details surrounding their relationships.	Pen and paper
The Pictorial Personality Traits Questionnaire for Children [36]	Self-report questionnaire that uses images to explore children’s personality traits, underpinned by the Big 5 Personality Traits theory.	Tablet
The approach-avoidance task [37]	A task used to index children’s approach and avoidance of emotional faces (angry vs happy).	Tablet
The Youth Life Orientation Test [38]	Self-report questionnaire assessing optimism and pessimism.	Tablet
The Scrambled Sentences Task [39,40]	A task used to assess interpretations of ambiguous sentences.	Tablet
Door Opening Task [41,42]	A computerized response perseveration task aimed at studying reward dominance in children.	Tablet
Words for Feeling Questionnaire	Used to measure children’s emotional vocabulary. Children are asked to come up with different synonyms of emotional words (eg, surprised, happy, scared).	Pen and paper

#### Parents’/Carers’ Measures

Table 3 shows the list of measures that parents/carers will complete about their children [43-49].

**Table 3:** List of measures that parents/carers will complete about children.

Name of the scale	Brief description	Style of administration
Demographics and socioeconomic status questionnaire	Questions will ask about the child’s date of birth, sex, ethnicity, country of birth, and number of siblings. We will also include questions about socioeconomic status, child’s religion, family migration status, family structure (ie, foster family, single parent, nuclear family), carers’ relationship to the child (ie, biological, foster or adoptive mother or father), household number, the child’s birth order, diet type they primarily follow, if the child has ever been in the care of local authority (ie, in foster or residential care), and if the child or anyone in their immediate family has ever been formally diagnosed with a mental health disorder. Socioeconomic status questions will include items relating to the carers’ education, occupation, material possessions, whether the child receives free school meals, type of accommodation, and post code (to ascertain material deprivation index).	Online platform
Patient Health Questionnaire 15 [43]	These measures link to physical health problems, including any chronic illness or disability, pain, fatigue, and sleep. For the Patient Health Questionnaire, we will only use questions relevant to physical health problems.	Online platform
Child Health Utility instrument 9D [44]	The Child Health Utility instrument is a pediatric generic preference-based measure of health-related quality of life—we will be using a proxy version for parents to complete. We will also be using questions formulated in-house to ascertain key factors such as hospitalization rates and birth weight.	Online platform
The Strengths and Difficulties Questionnaire [45]	The questionnaire asks about the parents or carers report on their child emotional symptoms, conduct problems, hyperactivity/inattention, peer relationship problems, and prosocial behavior.	Online platform
Arts Council Questionnaire The Taking Part Survey [46]	The survey collects information about children and adults’ attendance at a wide variety of arts events, museums, galleries, libraries, and heritage sites. It also asks about participation in creative activities and sport and motivations and barriers to engagement.	Online platform
The Children’s Life Events Inventory [47]	Questionnaire used to identify the number of significant life events experienced by the child.	Online platform
The Parenting Styles and Dimensions Questionnaire [48]	Questionnaire used to assess parenting styles that we will be using in the short version of this scale. It measures 3 types of parenting styles: authoritative, authoritarian, and permissive parenting.	Online platform
Parental report of their children social media usage	Questionnaire asking about children’s social media use.	Online platform
The Child version of the Eating Disorder Examination [49]	The Child version of the Eating Disorder Examination questionnaire provides assessment of eating disorder psychopathology related to anorexia nervosa, bulimia nervosa, and binge-eating disorder; however, the Child version of the Eating Disorder Examination questionnaire-8 items does not assess symptoms of avoidant/restrictive food intake disorder, pica, or rumination disorder. Originally, this is a self-report questionnaire, but we will use it as a parent/carer report format.	Online platform

#### Teachers’ Measures

Table 4 [45,50] shows the list of measures that teachers will complete about their children.

**Table 4:** List of measures that teachers will complete about children.

Name of the scale	Brief description	Style of administration
The Strengths and Difficulties Questionnaire [45]	The questionnaire asks about the teacher’s report on students’ emotional symptoms, conduct problems, hyperactivity/inattention, peer relationship problems, and prosocial behavior.	Pen and paper
Amended: Questionnaire about school resources for mental health support [50]	The questionnaire will ask about all the different resources available to students that are provided by the school for mental health support. Questions about creative arts therapies have been added.	Online platform

### Data Analysis

#### Planned Analyses

This study will explore the association between mental health and resilience in primary school children while assessing the impact of the psychological factors, social factors, and resources available to them. We will also assess the resilience scores in this age group, the psychometric properties, and how these scores might predict mental health problems in relation to the psychological factors, social factors, and resources available. As our dataset will contain multiple possible combinations of the predictors and different ways of operationalizing resilience (even across time), more specific hypothesis-driven analysis will be individually preregistered on the Open Science Framework website prior to any analysis. A key analysis will include assessing the impact of the opt-in versus opt-out recruitment process on systematic selection bias. Where relevant and if this overall analysis is significant, we will include this as a sensitivity analysis on a paper-by-paper basis, and this will be stated in the analysis preregistration.

To assess the feasibility (uptake, retention) of developing a longitudinal cohort study of preadolescent children in East London, we will record information on our school recruitment process: (1) number of schools contacted, (2) number of schools who responded with interest, (3) number of schools who had an initial meeting with us, (4) number of schools where we reached out to parents/carers, (4) number of schools where we reached out to children, (5) number of children we spoke to, (6) number of parents/carers who provided consent, (7) number of children who provided assent, (8) number of children who completed at least one assessment, (9) number of parents/carers who completed at least one assessment, and (10) mean number of assessments completed.

#### Missing Data

Where data are missing, these will either be imputed, or the participant will be removed from the analysis as appropriate for the statistical test being conducted. Because this method will vary according to the nature of the hypotheses and the statistical tests being conducted for any one individual paper, it cannot be stated now, but this will be included in the Open Science Framework preanalysis registration as appropriate (on a paper-by-paper basis). As there is no source documentation for the measures collected directly onto tablets, data cannot be entered during quality assurance checks. Data entry rules will make it very difficult for data to be missed during entry.

## Results

Barts Charity awarded a grant to the Faculty of Medicine and Dentistry, Queen Mary University of London, on March 30, 2020, to establish the Youth Resilience Unit. The Unit was established on March 1, 2021. This core funding also provided support for this study. Ethics approval (QMERC22.251) was granted by the Queen Mary research ethics committee on June 27, 2022. Study recruitment commenced from October 2022 and continued till December 2023. Baseline testing commenced in October 2022 and continue till December 2023; 873 students were enrolled at baseline. Follow-up is anticipated to continue at least annually until June 2027. Baseline and follow-up questionnaires will provide evidence regarding the acceptability and feasibility of conducting a longitudinal cohort study in primary schools in East London.

## Discussion

### Overview

Resilience is the ability to overcome adverse events and experience positive outcomes. Although the development of emotional resilience has been explored in adolescence, there is a dearth of evidence on the nature of and development of resilience among preadolescent populations.

The DEER study aims to explore how preadolescent children define resilience, track the development of resilience, and asses the contributory factors to differences in resilience pathways across time. It is hoped that the results of our study will allow us to work on more developmentally sensitive theories of resilience and propose future interventions to reduce negative outcomes following adverse events in childhood. The core analysis of the study will focus on investigating the trajectories of resilience across time and exploring its links with mental and physical health and other child characteristics. However, as the planned analysis is highly flexible and responsive to changes in the field, it is not possible to speculate meaningfully about this here, but all analyses will be registered on the Open Science Framework website prior to any analysis being conducted.

### Strengths of This Study

We believe this is one of the first longitudinal cohort studies exploring the factors associated with the development of emotional resilience in primary school-age children. In our measures, we also assess multiple risk and protective factors from individual traits (including cognitive information processing and lifestyle factors) to social and community resources. We also employ mixed methods, including quantitative and qualitative data, as well as experimental methods in cognitive tasks.

This provides a unique opportunity to understand the factors involved in the early stages of how resilience emerges and develops as well as the impact of these various factors over time. Our sample is also notable in being one that includes children growing up in socioeconomically deprived and marginalized communities. These children are not only typically underrepresented in mental health research, but they may also face multiple chronic stressors (poverty, housing difficulties, discrimination) where resilience—individual traits and external resources—are especially relevant in navigating these daily challenges. The flexible nature of the cohort measures allows us to respond to early findings and new information or data in the field as this is published.

### Weaknesses in This Study

This study relies heavily on self-report from young children, and it is not always clear how reliable or valid this method is in children of this age. We have tried to balance this by including measures by teachers and parents. However, parents are notoriously difficult to engage in this process, and this may result in lower levels of data from parents. Teachers also may be less responsive due to their many responsibilities and limited time to complete assessments. The large number of child assessments may also cause participant fatigue, particularly in a school setting. This may lead to nonrandom missingness in the results.

### Dissemination Plan

The results of this study will be published in academic journals and presented at academic conferences throughout the life of the project. We will also use the Youth Resilience Unit and the Unit for Social and Community Psychiatry social media accounts to further disseminate the research results. A brief debriefing report will be prepared for all participants, parents/carers, and schools, which will be circulated when the results have been prepared. Oral presentations of the key findings will also be offered to all participants, parents/carers, and schools.

All dissemination activities will draw on established research and clinical networks in our partners. Dissemination will be conducted with consideration of the safe data and safe statistical analysis protections. No individual level data will be published that could be linked back to a person or institution, and all output will be assessed to determine if there is a risk of disclosure through things such as single frequency counts in tables or identifying information in any qualitative fragments. In such cases, data will be censored, obscured, or changed to protect individual confidentiality. Where possible, any sensitive or difficult findings will be discussed with the participants or relevant institution if this is about a clinic or similar setting, prior to publication to avoid any inadvertent reputational harm or negative consequences of the findings being made public.

## Abbreviations

DEER: Development of Emotional Resilience
REDCap: Research Electronic Data Capture

## Data Availability

Fully anonymized data arising from the study and any other study materials will be available on reasonable request from the study principal investigators (j.lau@qmul.ac.uk and d.ougrin@qmul.ac.uk) governed by a signed data sharing agreement after the publication of the findings of the study by the research team.
